# A prospective study investigating the efficacy and toxicity of definitive ChemoRadiation and ImmunOtherapy (CRIO) in locally and/or regionally advanced unresectable cutaneous squamous cell carcinoma

**DOI:** 10.1186/s13014-021-01795-5

**Published:** 2021-04-09

**Authors:** Charles Lin, Trishna Ballah, Michelle Nottage, Karen Hay, Benjamin Chua, Lizbeth Kenny, Paul Thomas, Michele Teng, Jacqui Keller, Trang Le, Jennifer Edmunds, Brett Hughes

**Affiliations:** 1 Department of Radiation Oncology, Royal Brisbane and Women’s Hospital, Butterfield Street, Herston, QLD Australia; 2 Department of Medical Oncology, Royal Brisbane and Women’s Hospital, Butterfield Street, Herston, QLD Australia; 3 Department of Nuclear Medicine, Royal Brisbane and Women’s Hospital, Herston, QLD Australia; 4 Queensland Institute of Medical Research, Berghofer Medical Research Institute, Herston, QLD Australia; 5 Faculty of Medicine, University of Queensland, St. Lucia, QLD Australia

**Keywords:** Skin cancer, SCC, Curative, Radiotherapy, Immunotherapy

## Abstract

**Background:**

Patients with unresectable advanced cutaneous squamous cell carcinoma (cSCC) are generally treated with palliative intent. Immune checkpoint blockade has significant activity in the palliative setting in patients with recurrent or metastatic cSCC. This single arm phase 2 prospective study aims to investigate the combination of curative intent chemoradiation and durvalumab (anti-PD-L1 checkpoint inhibitor) for this patient cohort.

**Methods:**

Patients with unresectable locally and or regionally advanced pathologically confirmed cSCC (stage III-IVa) deemed fit for CRIO by consensus of the Multidisciplinary meeting will be eligible. In the first stage of a two-stage minimax design, we aim to recruit a total of 15 patients. If fewer than 7 patients achieved a complete response in the first stage, we will conclude the treatment is not more effective than standard treatment. The co-primary endpoints of CRIO are the safety of treatment (acute and late toxicities) and the rate of complete response. Secondary endpoints would include overall survival, progression free survival, and locoregional control. Translational research endpoints including biomarkers (CD73, CD39, PD-1, PD-L1) will also be explored utilising multiplex immunohistochemistry on tumour biopsy samples obtained prior to commencing treatment and during treatment (week 2). In addition, the utility of CXCR-4 PET/CT scan will be explored.

**Discussion:**

CRIO is a novel trial evaluating the combination of curative intent chemoradiotherapy with concurrent and adjuvant durvalumab for patients with unresectable stage III-IVa cSCC.

*Trial registration*: Trial registered with the Australian New Zealand Clinical Trial Registry (ACTRN12618001573246)

## Background

Cutaneous squamous cell carcinoma (cSCC) of the skin is the second most common skin cancer (after basal cell carcinoma). Lifestyle changes in the last 50 years have led to increased exposure to sunlight provoking a sharp rise in its incidence worldwide. Australia has one of the highest incidence of cutaneous skin malignancies in the world [1]. Early cSCC is often curable with local therapy. Metastases are rare, but spread to regional lymph nodes or more distant sites occurs in 5–10% of patients and confers a poor outcome [2, 3]. Presentations with locally advanced primary disease and/or regional nodal metastases (Stage III and IVa, AJCC 8th edition) [4] is not uncommon and patients face the grim prospect of morbid surgery or uncontrolled loco-regional disease affecting critical areas in the head and neck. Current standard of treatment is surgery followed by adjuvant radiotherapy. However, a proportion of patients cannot undergo surgery due to significant medical comorbidities which put them at high risk of intraoperative mortalities. This group of patients are categorised to have medically unresectable disease. Some patients have diseases that the MDT consensus is that R0 resection is unlikely to be achieved. For example, big nerve perineural diseases closely related to critical organs such as spinal cord or brain stem; extensive dermal spread of cSCC; nodal diseases encasing the carotid arteries. Some patients refuse to undergo surgery due to the consequential functional and/or cosmetic morbidities following total rhinectomy and/or orbital exenteration or other forms of extensive cranio-facial resections. For the purpose of this study, patients refused to have mutilating surgery and/or residual diseases anticipated due to proximity to critical organs at risk are categorised to have surgically unresectable disease.

Patients with stage III-IVa unresectable diseases can be treated with single modality radiotherapy or immunotherapy. It is anticipated that locoregional control and cure rates would be very low with single modality radiotherapy [5].

The use of concurrent chemotherapy with radiotherapy for cSCC in the definitive setting has been investigated in several retrospective series, with most utilising cisplatin-based chemotherapy. Results indicated partial response between 17 and 54% and complete responses in 17 to 43% [6–9]. Our institution has published the only prospective study of definitive chemoradiation in this setting. This demonstrates a 50% rate of complete response with combined radiotherapy and concurrent cisplatin/carboplatin [10]. Complete responders after definitive chemoradiation rarely recur in the irradiated area. It is therefore logical to consider adding an agent to improve on the 50% complete response rate.

cSCC has several clinical and biological factors which suggests it is appropriate for the clinical study of inhibition of the Programmed Death-1/Programmed Death-Ligand 1 (PD-1/PD-L1) immune check point: high mutation burden [11] presence of tumour-infiltrating-lymphocytes [11–14], association with immunosuppression as a risk factor [15], evidence of direct immunosuppressive effects of UV radiation [16], and some clinical efficacy with interferon 2α-based treatment [17]. The presence of high mutation burden is also a common characteristics of other solid tumours for which inhibition of the PD-1/PD-L1 axis has been associated with therapeutic efficacy, including melanoma, non-small cell lung cancer, and urothelial cancer [18].

The use of PD-1/PD-L1 immune check point inhibitor is generally well tolerated and publications of prospective phase 2 studies [19, 20] on cemiplimab have shown impressive response of patients with recurrent advanced/metastatic cSCC being sensitive to PD-1 blockade. The survival benefit of adding immunotherapy to chemoradiation is also evident in the setting of locally advanced non-small cell lung cancer as shown in a randomized controlled trial [21]. Durvalumab (MEDI4736) is a human monoclonal antibody of the immunoglobulin G-1 kappa subclass that inhibits binding of PD-L1. As Durvalumab (MEDI4736) is an engineered monoclonal antibody, it does not induce antibody-dependent cellular cytotoxicity or complement-dependent cytotoxicity.

We hypothesize the addition of durvalumab will improve the rate of complete response to definitive chemoradiation for patients with unresectable stage III-IVa cSCC. The CRIO study aims to primarily investigate and report the efficacy (rate of complete response) and safety (incidence of treatment related toxicities) of this novel therapeutic approach. The rate of progression free survival, locoregional control, and overall survival will also be reported as the secondary outcome.

Translational research endpoints including biomarkers will also be explored utilising multiplex immunohistochemistry on tumour biopsy samples obtained prior to commencing treatment and during treatment (week 2). In addition, the utility of CXCR-4 PET/CT scan will be explored. Participation in the translational study is optional. Similarly, participation in the CXCR-4 PET/CT study will also be optional. Patients will receive the same treatment regardless of their participation in the translational and CXCR-4 PET/CT sub-studies.

### Objectives

The primary objectives were to

1. Assess the safety of treatment (treatment related acute and toxicities) and to
2. Assess the rate of complete response.

The secondary objectives were to assess overall survival, progression free survival, and locoregional control. In addition, the correlation of CXCR-4 uptake on PET/CT scan (see below in Study setting) to overall survival, progression free survival, and locoregional control.

## Methods/design

CRIO is a single arm, non-blinded, prospective, phase II study with a two-stage minimax design in a single Australian academic hospital.

### Methods: participants, interventions and outcomes

#### Study setting

This trial is a single arm, phase II, prospective study conducted in a single Australian academic hospital in Queensland. Data will be collected at the hospital where patients are being accrued.

#### Translational studies

Adenosine is generated in response to proinflammatory stimuli such as cellular stress initiated by hypoxia or ischemia [22, 23]. Release of extracellular ATP undergoes conversion to AMP by the enzyme CD39 and subsequent dephosphorylation of AMP to adenosine is catalyzed by CD73. Adenosine signaling, particularly via the A2A adenosine receptor, potently reduces effector functions of cytotoxic lymphocytes (CD8+ T cells and NK cells) while also promoting recruitment and polarization of immunosuppressive cell types, including myeloid-derived suppressor cells and T regulatory cells [24]. Previously, we and others have identified that targeting adenosine generation by blockade of the ectonucleotidase CD73 or downstream A2AR inhibition enhances tumour control and anti-metastatic activity [25–28]. In addition, therapeutic approaches targeting the adenosinergic pathway alongside immune checkpoint blockade and chemotherapies display enhanced anti-tumour efficacy in combination [22, 29–31]. Importantly, targeting adenosine in solid tumours using anti-CD73 (NCT02503774) or A2A adenosine receptor antagonism (NCT02403193 and NCT02655822) has entered clinical trials.

We previously demonstrated that in more advanced clinical stage, CD73 expression is increased in cutaneous melanoma patients who did not receive any immunotherapy or BRAF/MEKi. In this present study, we aim to evaluate if the adenosinergic pathway is present in cSCC and its relationship with the PD-1/PD-L1 pathway. We will utilize multiplexed immunohistochemistry to evaluate the expression of CD73, CD39, PD-1, PD-L1 on tumour, endothelial or immune cells on tumour biopsies to examine cellular aspects of response. These biopsies will be obtained at baseline and if feasible, biopsies will also be obtained at week 2 while patients are on treatment. Furthermore, all patients will have serial blood sampled (baseline, weeks 3, 13, 21 and 37) for retrospective immunological and molecular studies should funding be obtained. This will include FACS analysis for innate and adaptive immune cell, and their expression of activatory and inhibitory receptors. mRNA can be isolated and immune signatures assessed using either a Nanostring panel or RNAseq. The clonality of T-cells can be examined by sequencing the TCR using Illumina-based next generation sequencing.

Results could provide the first proof-of-principle clinical evidence of the prognostic relevance of the density of immune cell infiltration, and presence of the adenosinergic pathway in cSCC.

Participation in the translational substudies is optional. Patients will receive the same cancer treatment regardless of their participation.

### CXCR4 PET/CT scan as a pilot sub-study

CXCR-4 is a chemokine receptor identified to promote the metastasis of a variety of cancer, including mucosal head and neck SCC. The over-expression of the CXCL12/CXCR4 chemokine system is likely to play a role in several tumour promoting effects including metastasis and resistance to treatment [32]. Promising human trial results for antibodies targeting other T-cell immunosuppressive mechanisms (e.g. immune checkpoints PD-1/PD-L1 and CTLA-4) have been reported for several cancers. A recent preclinical study demonstrated that targeting CXCR4 through inhibitory antibodies, in combination with anti-PD-L1, caused tumour regression in a mouse model of cancer which is resistant to PD-1 and CTLA-4 blockade [33]. This provides a good rationale for exploring the relationship between CXCR4 and immunotherapy, of which little is known in human SCC.

At the Herston Imaging and Research Facility within our institution, we have access to a novel Positron Emission Tomography (PET/CT) tracer, 68Ga-CPCR4-2 (68Ga-Pentixafor) which binds to CXCR4 receptors. It is hypothesized that cutaneous SCC with higher CXCR4 uptake would be at greater risk of nodal and distant metastases. As a pilot sub-study, we aim to explore the utility of CXCR4 in predicting the response to CRIO. Participation in the CXCR4 pilot sub-study is optional and all patients enrolled in the CRIO study will be treated and followed up the same way. The information obtained from the CXCR4 pilot study is hypothesis generating given the likely small patient number. If a trend is detected in its predictive utility, this may lead to the use of CXCR4 PET/CT for cSCC in future trials.

Participation in the CXCR-4 PET/CT substudy is optional. Patients will receive the same cancer treatment regardless of their participation.

#### Eligibility criteria

Inclusion criteria were as follows: adult patients presenting with pathologically confirmed unresectable* cSCC with locally-advanced disease at the primary site with or without metastases to the nodal regions, or advanced regional lymph node metastases of presumed skin origin, or unresectable perineural disease (stage III-IVa); Eastern Cooperative Oncology Group performance status 0–2; and assessable disease. Patients with bulky disease are assessed using RECIST criteria. Patients with perineural disease must have disease visible on imaging or biopsy-proven disease.

Exclusion Criteria include: Immunosuppression secondary to haematological malignancy or the use of immunosuppressant following solid organ transplantation; active infection requiring therapy with HIV, Hepatitis B or C virus; active uncontrolled bleeding; previous chemotherapy and/or radiotherapy to the head and neck region which would preclude re-treatment; contra-indication for radiotherapy or chemotherapy; claustrophobia; previous treatment with a PD1 or PD-L1 inhibitor; receipt of anticancer therapy 60 days prior to the first dose of study drug; use of immunosuppressive medication within 14 days before the first dose of Durvalumab (MEDI4736); any unresolved toxicity NCI CTCAE v 4.03 Grade ≥ 2 from previous anticancer therapy; history of allogenic organ transplantation; active or prior documented autoimmune or inflammatory disorders and receipt of recent live attenuated vaccine.

*The definition of unresectable disease:

1. Medically unresectable: Patients likely to have high rate of intra/peri-operative morbidities or mortalities seconcry to significant medical comorbidities
2. Surgically unresectable as per the sub-specialty MDT consensus that R0 resection is unlikely to be achieved. Examples:
  Patients with diseases closely abutting critical organs at risk such as brainstem and/or spinal cord as in the setting of big nerve perineural spread;
  Patients with rapid dermal metastases at presentation where clear margins can not be safely achieved and/or disease is expected to recur rapidly after surgery;
  Patients with diseases abutting critical vasculatures (e.g. carotid artery) where residual disease is anticipated or risk of peri-operative morbidities/mortalities are considered to be high;
3. Patients refuse to undergo potentially mutilating cranio-facial resection due to the consequential functional and cosmetic morbities (e.g. orbital exenteration, total rhinectomy, complete facial nerve sacrifice etc.)

#### Who will take informed consent?

The informed consent process will be carried out either by the treating clinician or an appropriately trained member of the site research team. A treating clinician will provide eligible patients with the information and consent form for the trial. Patients will be given time to read the information and will be provided opportunities to clarify any concerns or ask questions prior to providing informed consent.

### Additional consent provisions for collection and use of participant data and biological specimens

Patients will be offered participation in two optional sub-studies. Patients will be asked to consent to participate in a sub-study which investigates the predictive power of a novel PET/CT scan using a novel CXCR4 tracer. Such a tracer has never been used in patients with cSCC in Australia. The result of this PET/CT scan will not impact on the treatment the patient receives, though the results obtained from this sub-study may be used to adjust treatment for future patients suffering from a similar condition. The CXCR4 PET/CT scans will be performed at baseline (prior to starting treatment) AND during week 3 of the treatment.

Patients will also be asked for a repeat core biopsy of the skin cancer or enlarged lymph node during the second week of treatment. Additional consent for this second biopsy will need to be obtained. The purpose is to identify any potential biomarkers in the biopsy specimen which may predict treatment response.

Participation in both sub-studies is optional. Patients who decline to participate in this sub-study will receive the same treatment as other study participants.

### Interventions

#### Explanation for the choice of comparators

Not applicable. This is a single arm study without a comparator.

#### Intervention description

1. Chemotherapy

Platinum-based chemotherapy will commence on either day 1–3 of the radiation and preferably repeated on the same day of each week. A maximum of 7 weekly doses will be given. In cases where the chemotherapy is delayed or ceased, the radiotherapy will continue according to protocol. The primary chemotherapy regimen is cisplatin 40 mg/m^2^/week. Carboplatin may be used for patients with renal or cardiac or hearing impairment, at the discretion of the treating physician. Full blood counts, ELFT, magnesium, and renal function tests must be performed within 48 h prior to each weekly chemotherapy dose.

2. Immunotherapy

Durvalumab will be supplied by AstraZeneca as a 500-mg vial solution for infusion after dilution. The solution contains 50 mg/mL durvalumab, 26 mM histidine/histidine hydrochloride, 275 mM trehalose dihydrate, and 0.02% weight/volume (w/v) polysorbate 80; it has a pH of 6.0. The nominal fill volume is 10.0 mL. Investigational product vials are stored at 2 to 8 °C (36 to 46°F) and must not be frozen. Drug products should be kept in secondary packaging until use to prevent excessive light exposure.

- Concurrent phase: administered every 4 weeks during chemoradiation (weeks 1 and 5).
- Adjuvant phase: same dose and frequency as concurrent phase in weeks 9,13 and 17. Up to 6 additional doses until the final disease status can be determined on PET/CT. Patients who achieve complete response or progressive disease will discontinue durvalumab immediately. Patients who undergo salvage surgery for residual disease will receive 6 more doses of durvalumab after surgery.
- Fixed dosing of 1500 mg every 4 weeks (approximate 20 mg/kg every 4 weeks)

3. Radiotherapy

Intensity Modulated Radiotherapy (IMRT) should be utilised on all patients in the study cohort.

### Target volumes and doses

#### Locally advanced disease without nodal metastasis

Gross disease at the primary site: Gross Tumour Volume (GTV) should include all the imageable/palpable extent of the gross disease. In-transit dermal metastases, if evident, are to be included in the GTV. A recommended 5 mm margin is to be added to the GTV to form the Clinical Target Volume (CTV) to include any microscopic disease. CTV will then be expanded by 5 mm to form the Planning Target Volume (PTV), to account for any setup error and patient movement. Where possible, the PTV is to receive a total dose of 70 Gy in 35 fractions over 7 weeks as per ICRU 83 [34]. The dose variation across the PTV should not exceed + 7% and -5% of the prescription point (ICRU reference point dose). Where the location of the PTV precludes delivery of 70 Gy due to surrounding normal tissue constraint, a lower dose of 66 Gy in 33 fractions over 6.5 weeks is acceptable.

#### Prophylactic nodal irradiation

All attempts are to be made to include the first lymphatic draining echelon in the radiotherapy field (e.g. to include the parotid region when the primary disease is located at the temple). As these sites may harbour subclinical/microscopic disease, a total dose of 54 Gy in 35 fractions over 7 weeks, as per ICRU 83, is to be delivered. The dose variation across the PTV should not exceed + 7% and − 5% of the prescription point (ICRU reference point dose). The anatomical boundaries of cervical lymph node levels are to be defined as per Gregoire et al. [35].

#### Locally advanced disease or occult primary disease with nodal metastases

##### Gross disease at the primary site and macroscopically involved nodes

GTV should include all the imageable/palpable extent of the disease at the primary (skin) site and the macroscopically involved lymph nodes. In-transit dermal metastases, if evident, are to be included in the GTV. A recommended 5 mm margin is to be added to the GTV to form the CTV to include any microscopic disease. CTV will then be expanded by 5 mm to form the PTV, to account for any setup error and patient movement. Where possible, the PTV is to receive a total dose of 70 Gy in 35 fractions over 7 weeks as per ICRU 83. The dose variation across the PTV should not exceed + 7% and − 5% of the prescription point (ICRU reference point dose). Where the location of the PTV precludes delivery of 70 Gy due to surrounding normal tissue constraint, a lower dose of 66 Gy in 33 fractions over 6.5 weeks is acceptable.

##### Prophylactic nodal irradiation

The subsequent lymphatic draining echelons next to the involved nodal region should be included in the radiotherapy field (e.g. to include level II to III ipsilateral cervical lymph nodes when the parotid region is clinically involved). As these sites may harbour subclinical/microscopic disease, a total dose of 54 Gy in 35 fractions over 7 weeks, as per ICRU 83, is to be delivered. The level of cervical lymph nodes is to be defined as per Gregoire et al. [35].

##### Large nerve perineural spread (PNS)

The extent of PNS can be defined on 3 T Magnetic Resonance Imaging (MRI) and/or clinical examinations. The most commonly involved cranial nerves in the setting of head and neck cutaneous SCC are the fifth (Trigeminal) and the seventh (Facial) cranial nerves. PNS are to be encompassed in the CTV and a 5 mm margin should be added to form the PTV. However, a smaller margin of < 5 mm can be accepted if vital organs at risk are immediately adjacent to the PNS. Where possible, the involved nerve(s) should receive ≥ 66 Gy in 33 fractions. Where vital organs at risk (e.g. optic chiasm or brain stem) are immediately adjacent to the PNS, a lower dose between 54 Gy − 66 Gy in 2 Gy per fraction can be accepted.

The extent of elective nerve irradiation in continuity with the known PNS disease (Based on MRI and/or clinical examinations) is to be determined by the treating Radiation Oncologist. For example, a patient with evidence of right infraorbital nerve involvement may also receive elective radiotherapy to the proximal segment of V2 up to the trigeminal ganglion. The dose of elective nerve irradiation is generally between 50–60 Gy, respecting the surrounding vital organs at risk.

All radiotherapy target volumes will be peer reviewed and approved by another head and neck and skin Radiation Oncologist prior to planning.

### Criteria for discontinuing or modifying allocated interventions

Intervention safety monitoring and assessment Adverse events (AE), defined as any untoward medical occurrence(s) in a trial participant regardless of causality with trial interventions, will be systematically monitored and recorded. These will be classified and graded according to the National Cancer Institute Common Terminology Criteria for Adverse Events version 4.03 (NCI CTCAE v4.03).

Serious AEs (SAE) will be reported to the appropriate ethics committees and authorities as well as the study safety committee. A suspected unexpected serious adverse reaction (SUSAR), which is an unexpected SAE related to the intervention, will additionally be reported to the drug manufacturer. If the AE is deemed by the investigator to have been caused, or probably caused, by the investigational treatment (denosumab), this will be labelled a treatment-related adverse event (TRAE). Study participants will be reviewed clinically for the presence of AEs by the site investigator prior to each cycle of chemotherapy or immunotherapy. Blood tests, including complete blood count, liver and renal function tests, electrolytes, thyroid function tests and serum cortisol will be reviewed at these visits. Immune-related adverse events are managed following algorithms as compiled by expert groups or as available in the product information. Re-challenge after a suspected TRAE is permitted provided symptoms resolve to an appropriate level to meet criteria to resume treatment and do not meet any of the permanent discontinuation criteria (such as grade 4 and select grade 3 toxicities). Dose reductions and dose escalations are not permitted. An independent data safety monitoring committee will monitor the conduct and safety of the trial during recruitment.

#### Permanent discontinuation of study treatment

In the event of an infusion reaction of grade ≥ 3 severity during or directly following durvalumab infusion, dosing should be stopped and the patient must permanently discontinue durvalumab treatment.

Study treatment will be permanently stopped in the event of evidence of pregnancy.

In addition, study treatment for any patient may be discontinued for other safety reasons or compliance issues at the discretion of the investigator or sponsor. A patient may choose to discontinue study treatment or study participation at any time for any reason.

A patient who permanently discontinued durvalumab treatment should continue follow-up in the study without additional treatment until progression of disease, completion of all study assessments, or closure of the study.

#### Management of infusion/allergic/hypersensitivity reactions

Acute infusion reactions are defined as any AE that occurs during the infusion or within 2 h after the infusion is completed. Emergency equipment and medication for the treatment of these potential adverse effects (e.g. antihistamines, bronchodilators, intravenous saline, corticosteroids, paracetamol and/or adrenaline] must be available for immediate use. Infusion reactions must be reported as AEs and graded according to the NCI-CTCAE version 4.03 grading scale.

#### Termination of the infusion

The infusion should be terminated and NOT restarted if any of the following AEs occur: anaphylaxis, laryngeal/pharyngeal oedema, severe bronchospasm, chest pain, seizure, severe hypotension.

### Strategies to improve adherence to interventions

Regular review by the treating medical team (Medical and radiation oncology] and manage any toxicities early. Adjuvant phase of immunotherapy following completion of chemoradiation will require careful coordination and support to overcome logistical difficulties particularly for patients from out of town.

### Relevant concomitant care permitted or prohibited during the trial

Standard concomitant care to support patients through CRIO will be allowed such as standard anti-emetics for cisplatin and appropriate supportive care for patients undergoing chemoradiotherapy.

### Provisions for post-trial care

Patients will be followed up for a total of 5 years from the time of diagnosis (minimum 2 monthly in the first year, 3 monthly in the second year, and 6 monthly from years 3–5). Patients’ treating general practitioners and community will also be engaged in ongoing care particularly in the immediate four to six weeks after chemoradiation when ongoing skin dressings may still be required on a frequent basis.

### Outcomes

#### Primary outcome

1. To assess the safety of CRIO-incidence of treatment related toxicities
  All treatment related toxicities will be recorded at each clinic visit for a total of two years in the Case Report Form using the CTCAE 4.03.
2. To assess the rate of complete response

##### Patients with measurable disease

**Fig. 1:**
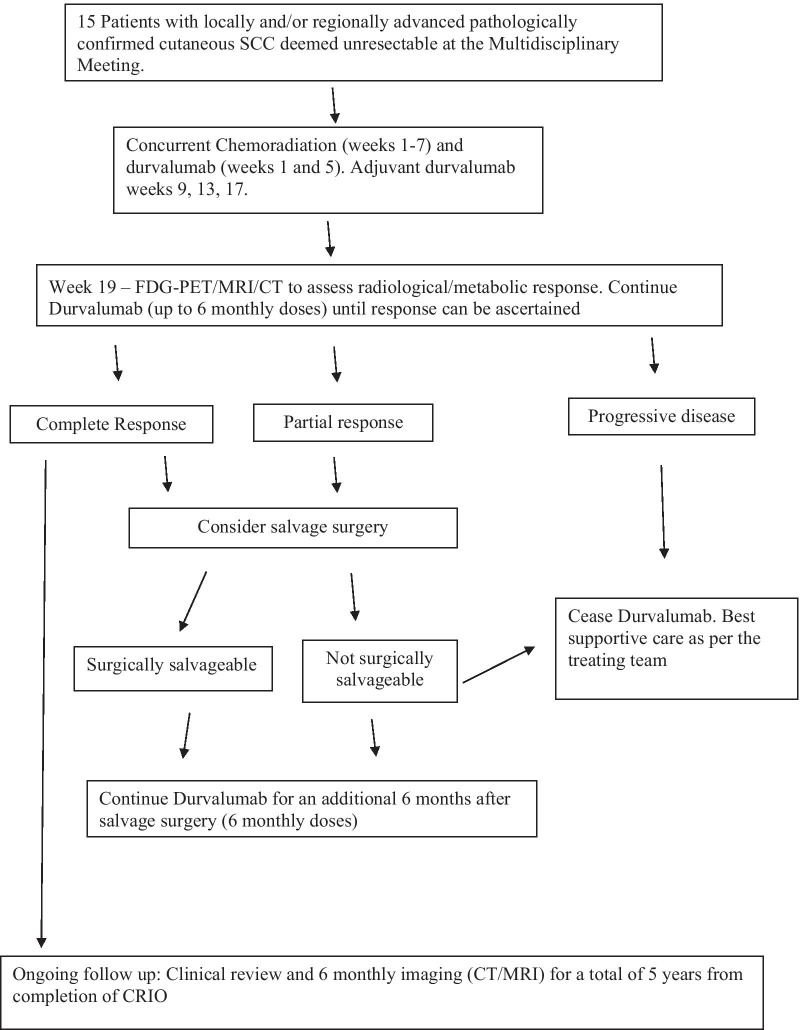
Consort diagram of CRIO trial

PET/CT scan +/− CT/MRI should be repeated at 19 (+/−2) weeks. Please refer to Fig. 1. Where results are equivocal, the clinician should either biopsy the suspected residual disease or repeat the imaging after an appropriate interval (week 27). The patient has achieved a CR if clinical and image findings are negative; if residual image findings are biopsy-negative; or if repeat imaging demonstrates resolution/stability of residual abnormalities.

Patients who do not achieve a CR should be considered for salvage surgery. Patients who achieve CR after such surgery will be designated CR (Su) and will undergo follow up as above. Patients with residual disease which is unresectable will be recorded as having a treatment failure.

##### Patients with large nerve perineural spread

These patients are not eligible for assessment of complete response and will be assessed as progression-free or not based on symptoms and/or image findings. Clinical follow-up should occur as above. The first progress MRI should be performed at 3 months from completion of treatment and then 6 monthly thereafter.

Once a CR has been declared, patients should cease the Durvalumab and clinical follow-up at four weekly intervals for a total of 1 year from the start of treatment. The follow up schedule can be found in Table 1. Physical examination will include direct inspection and palpation of accessible sites. Imaging will be done if there is clinical suspicion of recurrence or progression. Biopsy or cytological confirmation of recurrent disease should be obtained whenever possible. The clinician will record disease status, sites and dates of any relapse and late effects of treatment.

**Table 1:** Schedule of assessments

Investigation	Screening /Baseline	Wk 1	Wk2	Wk3	Wk4	Wk5	Wk6	Wk7	Wk9	Wk13	Wk17	Wk 19	Wk 21, 25, 29, 33, 37, 41	Wk 27	Months post treatment 2,4,6,8,10,12,15,18,21,24,30,36,42,48,54,60
Informed consent	x														
Histological confirmation of SCC	x														
Repeat biopsy (skin or lymph nodes]			× ^1^												
Medical history	x^6^														
Physical examination (ECOG, vital signs, toxicity]	x^6^	x	x	x	x	x	x	x	x	x	x		x		x
Concomitant meds	x														
Urine analysis	x														
Pregnancy test	x^6^														
FBC	x^6^	x	x	x	x	x	x	x	x	x	x		x		x^4^
U&E, LFT’s	x^6^	x	x	x	x	x	x	x	x	x	x		x		x^4^
Coagulation profile, TSH, hep, HIV															
x															
FDG PET	x^7^											x		x^2^	
CXCR4 PET /CT	x^3^			x											
Contrast CT / MRI	x^7^											x^4^			
Disease status		x	x	x	x	x	x	x	x	x	x		x		x
Blood	x			x						x			x^5^		

^1^Not mandatory

^2^If week 19 FDG PET is equivocal

^3^Pretreatment

^4^If clinically indicated

^5^Weeks 21 and 37 only

^6^Due within 14 days of trial registration

^7^Due within 28 days of trial registration

Patients who are considered treatment failures will be followed at the above time points for survival only. This may be done at clinic visits when seen for ongoing treatment or by telephone call

### Definition of treatment failure

The patient will be considered as having treatment failure if:

- Progressive disease at any time;
- Residual disease at 12 weeks and not amenable to salvage surgery;
- Recurrent disease within the irradiated field during follow up that is biopsy-proven or increases over serial imaging/clinical examination;
- Metastatic disease

Secondary Outcomes include overall Survival, progression free survival, and locoregional control will be assessed.

### Participant timeline

A timeline of 2 years is used to accrue 15 patients. Please also see Table 1, schedule of assessments.

#### Sample size

A two-stage minimax design will be applied, which requires an initial sample of 15 patients, with possibly an additional 13 patients required (total 28) subject to feasibility. The hypothesis that the proportion responding is 70% or more compared to the alternative hypothesis that 50% or fewer respond will be tested using a two sided binomial test with a target significance level of 0.10 and power of 0.80. Fifteen patients would be recruited in the first stage and if 7 or fewer patients respond the trial will be terminated and we will conclude that the treatment is not more effective than standard treatment. If 8 or more patients respond, the trial may go to the second stage, recruiting a further 13 patients. Of the total of 28 patients, 18 or more must respond to the treatment to be considered better than current treatment. Sample size was calculated using the PASS 13 Power Analysis and Sample Size Software (NCSS, 2014).

#### Recruitment

Participants will be identified through specialist referral and through oncology multidisciplinary team meetings. Plastic surgeons and head and neck surgeons are well informed of the trial in our institution as most referrals would come from them. We will also advocate for patients to be treated with CRIO if surgery entails mutilating surgery such as cranio-facial resection which will result in significant functional and cosmetic morbidities.

### Assignment of interventions: allocation

#### Sequence generation

Not applicable. All patients in this trial will receive the same treatment.

#### Concealment mechanism

Not applicable. All patients will receive the same treatment.

#### Implementation

Not applicable. All patients will receive the same treatment.

### Assignment of interventions: blinding

#### Who will be blinded

Not applicable. All patients will receive the same treatment.

#### Procedure for unblinding if needed

Not applicable. All patients will receive the same treatment.

### Data collection and management

#### Plans for assessment and collection of outcomes

As per schedule of assessments (Table 1). All treatment related toxicities will be recorded at each clinic visit for a total of two years in the Case Report Form using the CTCAE 4.03. (https://evs.nci.nih.gov/ftp1/CTCAE/CTCAE_4.03_2010-06-14_QuickReference_8.5x11.pdf).

Disease status of patients will be recorded at each visit during and after completion of treatment.

#### Plans to promote participant retention and complete follow-up

Prior to study entry screening will be conducted by trial staff and will include consideration of the ability for the participant to complete treatment and follow-up. A participant is free to withdraw from this project at any stage and have the option to request for trial biological samples to be disposed of.

#### Data management

Only re-identifiable trial data will be stored in a password protected trial database. Trained, registered personnel will have access to the trial database via password protected login. All hardcopy (paper] data will be kept behind locked doors or in locked cabinets in the trial coordinator's room.

### Confidentiality

#### Patient confidentiality

The Investigator must ensure that the patient's privacy is maintained. A patient should only be identified by the unit code and his / her patient identifier on the case report forms or other documents submitted to the Data Agency. Only re-identifiable trial data will be stored.

#### Plans for collection, laboratory evaluation and storage of biological specimens for genetic or molecular analysis in this trial/future use

Translational studies will include analyses of tumour tissue and peripheral blood samples from participants (the biospecimens). This includes formalin-fixed, paraffin-embedded tumour tissue obtained at baseline (pre-treatment) and peripheral blood before and/or after treatment. Patients will be invited to undergo a repeat core biopsy of the gross disease at 2 weeks into the treatment. Participation for the repeat biopsy is optional. The rationale for the repeat biopsy is to explore if there are any interim biomarkers with treatment response. Peripheral blood will be transported to the central laboratory within 1 h of collection and processed with serum and peripheral blood mononuclear cells stored for future analysis when the trial is complete. Immune markers of interest will be assessed via multiplex immunohistochemistry for formalin-fixed, paraffin-embedded tumour samples or flow cytometry on peripheral blood mononuclear cells. The peripheral blood mononuclear cells will be stored in liquid nitrogen.

### Statistical methods

#### Statistical methods for primary and secondary outcomes

For the first stage, the analysis will focus on descriptive statistics. Categorical variables will be summarised using frequencies and percentages and continuous variables will be summarised using means with standard deviations or as medians with interquartile ranges. Median survival time (with 95% CI) will be described and survival time will be presented using a Kaplan–Meier curve. Safety/toxicity data will be presented according to the frequency of occurrence. The proportion of patients experiencing the primary outcome (complete response) will be presented with 95% confidence intervals and compared to the hypothesized null effect of 0.5 using an exact binomial test.

Secondary outcomes including overall survival, disease free survival, time to progression and loco-regional control will be presented using a Kaplan–Meier curve.

#### Interim analyses

No interim analysis is planned. The sample size in this study is small.

#### Methods for additional analyses (e.g. subgroup analyses)

No additional analysis is planned.

#### Methods in analysis to handle protocol non-adherence and any statistical methods to handle missing data

The primary analysis will be intention-to treat, with all patients included, regardless of adherence to the protocol. A per protocol analysis will be conducted as a sensitivity analysis for the proportion of patients showing CR.

#### Plans to give access to the full protocol, participant level-data and statistical code

Not applicable. The protocol is being submitted for consideration of publication. The results of the trial, when completed, will be reported and submitted for publication in appropriate peer reviewed journals.

### Oversight and monitoring

#### Composition of the coordinating centre and trial steering committee

Metro North hospital and health service (Queensland, Australia) has the overall responsibility to initiate and conduct the CRIO study and has designated responsibility to the Principal Investigator for the day-to-day conduct of the study and to lead, train and supervise study site staff. Astra Zeneca will collaborate to support the study as detailed in the research agreement.

This trial is reviewed by a data safety monitoring committee every 6 months to review safety and efficacy aspects of the trial,

#### Composition of the data monitoring committee, its role and reporting structure

This study has been reviewed and approved by the local institution’s Human Research Ethics Committee (HREC) and will be carried out according to the National Statement on Ethical Conduct in Human Research (2007). It is registered with the Therapeutic Goods Administration, under the Clinical Trial Notification scheme and will be conducted with reference to Guidance on Good Clinical Practice. This trial is also listed on the Royal Brisbane Hospital Risk Register and will be reviewed by a data safety monitoring committee every 6 months to review safety and efficacy of the trial.

All the above groups are independent of the sponsor and have no competing interests.

Any recommendations from the safety committee, annual reports and all SAE will be reported to the local institution’s HREC.

#### Adverse event reporting and harms

This trial is reviewed by a data safety monitoring committee every 6 months to review safety and efficacy aspects of the trial.

Any recommendations from the safety committee, annual reports and all SAE will be reported to the local institution’s HREC.

#### Frequency and plans for auditing trial conduct

This trial is listed on the Royal Brisbane Hospital Risk Register and will be reviewed by a data safety monitoring committee every 6 months to review safety and efficacy of the trial.

Any recommendations from the safety committee, annual reports and all SAE will be reported to the local institution’s HREC.

The Investigator will be responsible for assuring that continuing review (at least once per year) of the study is performed by the HREC throughout the duration of the study.

### Plans for communicating important protocol amendments to relevant parties (e.g. trial participants, ethical committees)

Approval will be obtained from the local institution’s HREC for any protocol amendments and amendments to the information and consent form for this study. Any amendments to the information and consent form also require the study participant to re-consent to participating in the trial.

#### Dissemination plans

The study results based on the trial data will be released to the participating physicians, referring physicians, patients and the general medical community. During study close-out, an interim period will be used to complete data collection, following which the manuscript(s) based on the trial results will be submitted to peer-reviewed journals. Authorship criteria as defined by the International Committee of Medical Journal Editors will be followed.

## Discussion

Patients with advanced cSCC in the head and neck region often require morbid deforming cranio-facial surgeries which often entail orbital exenteration and/or rhinectomy and/or complete facial nerve sacrifice. We have published the outcome of a similar patient cohort treated with definitive chemoradiation which reported 50% rate of complete response [10]. The addition of durvalumab to chemoradiation in this cohort of patients can potentially improve the rate of complete response. By eliminating the need for aggressive mutilating surgery, patients were able to live a more normal life with better functional and cosmetic outcomes. The CRIO study aims primarily to investigate the efficacy (rate of complete response) and the safety (incidence of toxicities) of this novel therapeutic approach for patients with unresectable cSCC.

This study also aims to explore potential molecular markers in identifying early responders or prognosticators. CXCR4-PET/CT and multiplex immunohistochemical tests were built in to identify potential good responders to CRIO.

## Trial status

Protocol version 1, dated 3 September 2018. The first patient was accrued on 09/05/2019. It is anticipated that recruitment will be completed in February 2022 although this timeline will be reviewed on a regular basis.

## Data Availability

The datasets used and/or analysed during the current study are available from the corresponding author on reasonable request.

## Abbreviations

cSCC: Cutaneous Squamous cell carcinoma
CRIO: ChemoRadiation and ImmuNotherapy
AE: Adverse event
ECOG: Eastern Cooperative Oncology Group
NCI CTCAE: National Cancer Institute Common Terminology Criteria for Adverse Events
PD-1: Programmed Death-1
PD-L1: Programmed Death-Ligand 1
CR: Complete response
SAE: Serious adverse event
SUSAR: Suspected unexpected serious adverse reaction
TRAE: Treatment related adverse event
UICC/AJCC: Union for International Cancer Control/
GTV: Gross Target Volume
CTV: Clinical Target Volume
PTV: Planning Target Volume
MRI: Magnetic Resonance Imaging
PNS: PeriNeural Spread
HREC: Human Research Ethics Committee

## Administrative information

Note: the numbers in curly brackets in this protocol refer to SPIRIT checklist item numbers. The order of the items has been modified to group similar items [see http://www.equator-network.org/reporting-guidelines/spirit-2013-statement-defining-standard-protocol-items-for-clinical-trials/]. Overall responsibility to initiate and conduct the study. The Institution has designated the Principal Investigator the day-to-day conduct of the study and to lead, train and supervise study site staff. Astra Zeneca will collaborate with the institution to support the study as per the terms in the research agreement
